# High Sucrose Ingestion during a Critical Period of Vessel Development Promotes the Synthetic Phenotype of Vascular Smooth Muscle Cells and Modifies Vascular Contractility Leading to Hypertension in Adult Rats

**DOI:** 10.1155/2022/2298329

**Published:** 2022-06-21

**Authors:** Vicente Castrejón-Téllez, María Esther Rubio-Ruiz, Agustina Cano-Martínez, Israel Pérez-Torres, Leonardo Del Valle-Mondragón, Elizabeth Carreón-Torres, Verónica Guarner-Lans

**Affiliations:** ^1^Department of Physiology, Instituto Nacional de Cardiología “Ignacio Chávez”, Mexico City, Mexico; ^2^Department of Cardiovascular Biomedicine, Instituto Nacional de Cardiología “Ignacio Chávez”, Mexico City, Mexico; ^3^Department of Pharmacology, Instituto Nacional de Cardiología “Ignacio Chávez”, Mexico City, Mexico; ^4^Department of Molecular Biology, Instituto Nacional de Cardiología “Ignacio Chávez”, Mexico City, Mexico

## Abstract

Cardiometabolic diseases, including hypertension, may result from exposure to high sugar diets during critical periods of development. Here, we studied the effect of sucrose ingestion during a critical period (CP) between postnatal days 12 and 28 of the rat on blood pressure, aortic histology, vascular smooth muscle phenotype, expression of metalloproteinases 2 and 9, and vascular contractility in adult rats and compared it with those of adult rats that received sucrose for 6 months and developed metabolic syndrome (MS). Blood pressure increased to a similar level in CP and MS rats. The diameter of lumen, media, and adventitia of aortas from CP rats was decreased. Muscle fibers were discontinuous. There was a decrease in the expression of alpha-actin in CP and MS rat aortas, suggesting a change to the secretory phenotype in vascular smooth muscle. Metalloproteinases 2 and 9 were decreased in CP and MS rats, suggesting that phenotype remains in an altered steady stationary state with little interchange of the vessel matrix. Aortic contraction to norepinephrine did not change, but aortic relaxation was diminished in CP and MS aortas. In conclusion, high sugar diets during the CP increase predisposition to hypertension in adults.

## 1. Introduction

Several effects of modified diets during the early stages of development on the susceptibility to develop hypertension during adult life have been reported, and it has been proposed that arterial hypertension may be determined by changes in environmental conditions during critical windows of development [1]. Many critical periods in development are present during the intrauterine period, infancy, childhood, and adolescence [2]. Alterations of the diet during gestation and lactation often result in low birth weight in offspring, which is related to a higher incidence of cardiometabolic and vascular diseases, including hypertension during adulthood [3–6]. A high sucrose diet before and during pregnancy and during lactation and the first days after weaning resulted in a higher incidence of hypertension when the rat offspring reached adulthood [7]. Similarly, a high salt diet during the same period of time also increased blood pressure [8]. Our group has described that a diet high in sucrose during a shorter critical window near weaning (rat postnatal days 12 to 28) that includes the last phases of lactation and the first days after weaning results in hypertension when the rats reach adulthood [9]. During this stage, the natural rodent diet changes from rich in fat to rich in carbohydrates, and there is important maturation of the endocrine pancreas. This process is accompanied by changes in glucose and insulin concentrations, which may modify vascular responses, and the effects are due to changes in the endothelin-1 release and are mediated through the ET_A_ and ET_B_ receptors [10, 11].

Furthermore, when sucrose is administered for a long period of time (24 weeks), the animals develop hypertension together with other signs of metabolic syndrome (MS). These animals also have alterations in vascular responses and hypertension [12]. In a previous paper by our group, we showed that, in this MS model, the levels of nitric oxide are decreased due to the decreased expression of endothelial nitric oxide synthase (eNOS) and the presence of oxidative stress in the thoracic aortas [9].

Hypertension is often related to changes in the phenotype of vascular smooth muscle cells (VSMC), which increase the stiffness of the vessel. VSMCs show different phenotypes during developmental stages, and these cells are even not terminally differentiated in adult organisms [13]. Their phenotype may change during different physiological and pathological conditions, and they may show a contractile or secretory phenotype. The contractile phenotype is characterized by a high concentration of markers such as alpha-actin, and the secretory phenotype is characterized by increased proliferation, secretion, and migration and decreased contractile markers [13]. Alterations in redox signaling [14] and inflammation [15] lead to changes in the VSMC phenotype, and glucose and insulin are associated with oxidative stress [14, 15]. Thus, redox homeostasis and inflammation may signal changes in the phenotype of smooth muscle cells [14] during the critical window near weaning.

Vascular remodeling is associated with metalloproteinases (MMPs) 2 and 9 that participate in the determination of the phenotype of VSMCs. These MMPs degrade the matrix that divides the media and the intima and allow for the migration of smooth muscle cells [16, 17]. Particularly MMP-2 and MMP-9 have been linked to the development of hypertension [18]. MMPs act as inflammatory cytokines during vascular formation or remodeling. The expression of active MMPs is absent or very low in mature and quiescent vessels. However, in tissues undergoing vascular remodeling, MMPs are markedly expressed, secreted, and activated [19].

In the present paper, we study changes in the morphology of the aorta that could increase the stiffness of the vessel and that correspond to differentiation to secretory VSMC phenotype. This change is usually accompanied by a decrease in the marker of the contractile phenotype alpha-actin. We further analyze changes in MMPs that may participate in the variation of the phenotype of VSMC. We compare these changes to the ones produced by long-term consumption of sucrose from weaning to seven months of age. We also evaluated the changes in vasoreactivity in adult rats that received sucrose during the critical window near weaning and in MS rats.

## 2. Materials and Methods

### 2.1. Animals and Experimental Groups

Experiments in animals were approved by the Laboratory Animal Care Committee of our institution and were conducted in compliance with our institution's ethical guidelines for animal research (INCAR Protocol number 20–1147).

Wistar male rats were given 30% sucrose in drinking water for different lapses of time: (a) during the postnatal critical period (postnatal days 12 to 28) (CP) and (b) MS animals that ingested sucrose solution during six months (including during the critical window). Control (C) rats that did not receive sucrose were also included. Three litters consisting of 8 male pups were used for each condition (24 rats for CP, 24 MS, and 24 C, total 72 animals).

In previous reports by our group where we used the animals from similar groups as those used in this study, animals were placed in metabolic cages to determine sucrose ingestion. In these previous studies, we demonstrated that there were no significant differences in the amount of water consumed among control, MS, and CP animals [9], and therefore, sucrose-fed rats ingested more kcal per day. All animals were fed Purina 5001 rat chow (Richmond, IN) ad libitum and were kept under controlled temperature and a 12 : 120 h light-dark cycle.

Rats were killed by decapitation after overnight fasting (12 h) after 6 months of treatment. The blood was collected, and the serum was separated by centrifugation at 600 g for 15 min at room temperature and stored at −70°C until needed. Thoracic aortas were obtained and cleaned from perivascular connective tissue and adipose tissue and frozen individually in Tissue Tek (Sakura Finetek Inc., Torrance, CA, USA) for histological analysis. For in vitro vasomotricity assays, the aortic rings were used immediately after extraction.

### 2.2. Biochemical and Physiological Determinations

Glucose concentration was assayed using an enzymatic SERA-PAK® Plus from Bayer Corporation (Sées, France). Serum insulin was determined using a commercial radioimmunoassay (RIA) specific for rats (Linco Research Inc., Missouri, USA); its sensitivity was 0.1 ng/mL, and intra- and interassay coefficients of variation were 5 and 10%, respectively. The HOMA-IR was calculated from the fasting glucose and insulin concentrations as previously reported [9].

Triglycerides were determined by commercially available procedures (Randox Laboratories Ltd., Antrim, United Kingdom). Endothelin 1 was quantified in serum by high-pressure liquid chromatography (UPLC), as previously reported [20]. Systolic arterial blood pressure was measured in conscious adult animals using the tail-cuff method; the cuff was connected to a pneumatic pulse transducer (Narco Bio-Systems Inc., Healthdyne Co.) and a programmed electrosphygmomanometer. The mean of five independent determinations was calculated.

### 2.3. Histological Analysis

Sections obtained from the thoracic aortic tissue from C, CP, and MS groups were embedded in paraffin (10 *μ*m) and processed and stained by the conventional methods for Masson's trichrome staining (TM) and Hematoxylin-Eosin (HE). In the TM images, the area of the vessel lumen, the total area of the media (Med), and the total area of the adventitia (Adv), as well as the thickness of the Med and Adv, were measured in images of the complete ring. To obtain these images, photographs were taken with the QImaging MicroPublisher 5.0 RTV (Real-Time Viewing) camera coupled to an Olympus microscope (BX51) at 10X, with the rear splice to complete the images of the rings (4 of each group, *n* = 4, total 16). The area measurements (pixels^2^) were determined with the Image-Pro Premier 9.0 program (Media Cybernetics, Inc., Rockville, MD, USA).

The analysis of the characteristics of the vascular wall was carried out from the HE stains by obtaining photographs of at least 4 fields of each tissue (*n* = 4 for each group). These photographs were obtained at a 40X magnification with a QImaging MicroPublisher 5.0 (Real-Time Viewing) RTV camera coupled to an Olympus microscope (BX51).

### 2.4. Western Blot Analysis

The aortas were homogenized in a lysis buffer, 25 mM HEPES, pH 8, 100 mM NaCl, 15 mM imidazole, 10% glycerol, 1% Triton X-100, 10 mg/ml deoxycholic acid, and protease/phosphatase inhibitor cocktail. The homogenate was centrifuged at 14,000 rpm for 15 min at 4°C; the supernatant was separated and stored at −70°C. The Bradford (Protein Assay, Bio-Rad Laboratories) method was used to determine the total proteins [21].

A total of 50 *μ*g protein was used and separated on an SDS-PAGE (12% bis-acrylamide-Laemmli gel) and transferred to a polyvinylidene difluoride (PVDF) membrane. Blots were blocked for 1 h at room temperature using Tris-buffered saline (TBS)-0.01% Tween (TBS-T 0.01%) plus 5% nonfat milk. The membranes were incubated overnight at 4°C with rabbit primary polyclonal antibodies *β*-actin (sc-81178), MMP-2 (sc-13595), MMP-9 (sc-393859), smooth muscle actin (SMA; sc-53142), and TNF*α* (sc-33639) from Santa Cruz Biotechnology (Santa Cruz, CA, USA). All blots were incubated with glyceraldehyde 3-phosphate dehydrogenase (GAPDH; sc-365062) antibody as a loading control. Images from films were digitally obtained by GS-800 densitometer with the Quantity One software (Bio-Rad Laboratories, Inc., Hercules, CA, USA), and they are reported as arbitrary units (AU).

### 2.5. Sample Preparation and Tension Recording

Aortas were immediately dissected after decapitation and placed in oxygenated normal Tyrode solution (mM: 140 NaCl, 5 KCl, 1 CaCl_2_, 1 MgCl_2_, 5 HEPES, and 5.5 glucose) pH 7.4. Arteries were carefully cleaned from connective and adipose tissue, taking care not to damage the endothelium. Tension measurements were made as previously described [22]. Briefly, segments of about 2 to 3 mm long were cut, and two 250 *μ*m diameter S-shaped silver wires (Medwire Corp) were inserted into the lumen to measure tension developed transversely by rings of the vessel. One of the silver wires was fixed to the bottom of an in vitro chamber, and the other was attached to a tension transducer that was connected to a Grass polygraph (model 79D, Grass Medical Instruments, Quincy, MA).

The chamber was filled with Tyrode solution, thermoregulated and bubbled with carbogen (95% oxygen, 5% carbon dioxide). A basal passive tension of 2 g was applied after determination in preliminary tests that this was the optimal resting tension under our experimental conditions. Arteries were allowed to rest for 1 h, and the solution was changed every 20 min. The contraction was induced twice by the addition of (a) 40 mmol/L KCl, (b) NE (1 *μ*M), (c) KCl plus insulin (50 *μ*U/mL), (d) KCl plus insulin and ET_A_ selective antagonist (BQ123 from Sigma Aldrich, San Luis, Missouri, USA) at 1 *μ*M, and (e) KCl plus insulin and ET_B_ selective antagonist (BQ788 from Sigma Aldrich, San Luis, Missouri, USA) at 1 *μ*M. Relaxation was induced by cumulative concentration-response curves to acetylcholine (Ach) 10^−9^–10^−4^ M on NE (1 *μ*M)-precontracted aortic rings. The EC50 and maximum dilation response (*E*_max_) values from the concentration-response curves of ACh for relaxation of the rat aorta were performed using the Sigma Plot (Systat Software, San Jose, CA, USA) program. The doses of KCl and NE were chosen after a dose-response curve. The KCl dose induced submaximal contractions that allowed us to observe elevations and decreases in tension developed. Arteries were washed by adding fresh Tyrode solution to the chamber allowing the rings to return to their basal tension (2 g). Mean contraction value was considered as 100% of response.

### 2.6. Statistical Analysis

Results are expressed as mean ± standard errors of the mean (SEM) from six different artery preparations. The percentage of contraction in each experiment was calculated, and the mean was then determined. Comparisons between groups were made by analysis of variance (ANOVA) followed by Student–Newman–Keuls, using the Sigma Stat program (Jandel Scientific, San Rafael, CA, USA). Differences were considered statistically significant when *P* < 0.05.

## 3. Results

### 3.1. Changes in Body Weight, Abdominal Fat, Blood Pressure, Glucose, Insulin, and Endothelin

The values of these variables in C animals that did not receive sucrose during development are shown in Table 1 and correspond to values of normal adult rats. Animals that received 30% sucrose in the drinking water during the CP showed similar values to those of C animals except for arterial blood pressure and serum endothelin levels, which were increased. Adult MS rats that received sucrose during and after the CP had body weight similar to C animals, but abdominal fat was significantly increased. They had hypertension, hypertriglyceridemia, hyperinsulinemia, and insulin resistance. Endothelin levels in serum in MS rats were also increased (Table 1).

### 3.2. Histological Changes in Aortas

**Figure 1:**
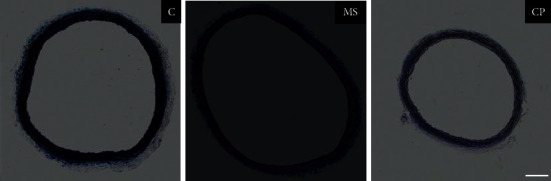
Representative images of the thoracic aortas stained with Masson's trichrome in the rats from the control (C), metabolic syndrome (MS), and critical period (CP) conditions. MS rats ingested sucrose solution during six months; CP animals ingested sucrose solution during postnatal days 12 to 28. Control rats received tap water. Bar = 100 *μ*m. There were no changes in the proportion of collagen fibers.

Images from the thoracic aortas from rats in the C, MS, and CP conditions stained with Masson's trichrome are shown in Figure 1. The average values of the total area of the lumen of the vessel and the Med and Adv are presented in Table 2.

**Figure 2:**
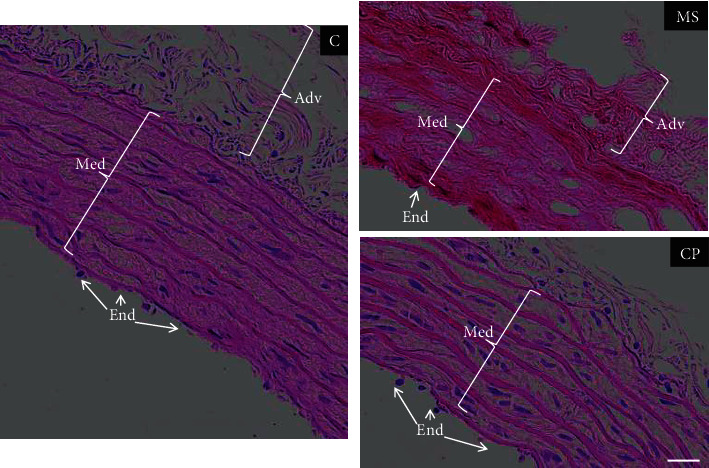
Photomicrographs of the rat thoracic aorta with HE staining under Control (C ), metabolic syndrome (MS) and critical period (CP) conditions. MS rats ingested sucrose solution during six months; CP animals ingested sucrose solution during postnatal days 12 to 28. Control rats received tap water. In C and CP the integrity of the tissue is observed with the endothelial cells (End) at the edge of the lumen of the vessel; elastic fibers and smooth muscle in the middle (Med) and adventitia (Adv). Bar = 25 *μ*m.

Regarding the organization of the layers in the vessels, we found that, in the C and CP, the layers were organized and well defined. There was integrity of the tissue with the endothelial cells at the edge of the lumen, elastic fibers, and smooth muscle in the Med and Adv. It should be noted that the endothelial lining is very fragile and is difficult to preserve in histological sections, which is evident for all three groups. In CP, some waviness of the elastic fibers is observed. In contrast, in the MS group, the organization of the vascular wall was lost, the endothelium (End) was disintegrated, the size of the Med and the Adv was reduced, cellularity was lost (no nuclei are observed in the smooth muscle), and empty spaces are shown indicating possible aneurysms (Figure 2).

### 3.3. VSMC Phenotype and Expression of Metalloproteinases and TNF Alpha

**Figure 3:**
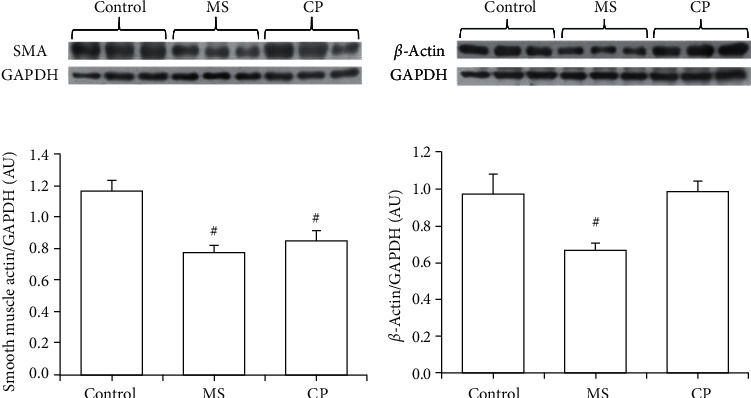
Expression of *α*- and *β*-actin in aortas from control, metabolic syndrome (MS), and critical period (CP) rats. MS rats ingested sucrose solution during six months; CP animals ingested sucrose solution during postnatal days 12 to 28. Control rats received tap water. A representative blot is shown above the graphics. SMA: smooth muscle actin; GAPDH: glyceraldehyde-3-phosphate dehydrogenase. There is a decrease in *α*-actin in both groups, and *β*-actin was only decreased in the MS group. Values are means ± SEM; ^#^*P* ≤ 0.05; *n* = 8.

Smooth muscle alpha-actin, which is a marker of the contractile phenotype of VSMC, was significantly decreased in MS and CP rats, thus indicating that, in these animals, the aortas show a secretory phenotype. In contrast, beta-actin was only decreased in MS rats (Figure 3).

**Figure 4:**
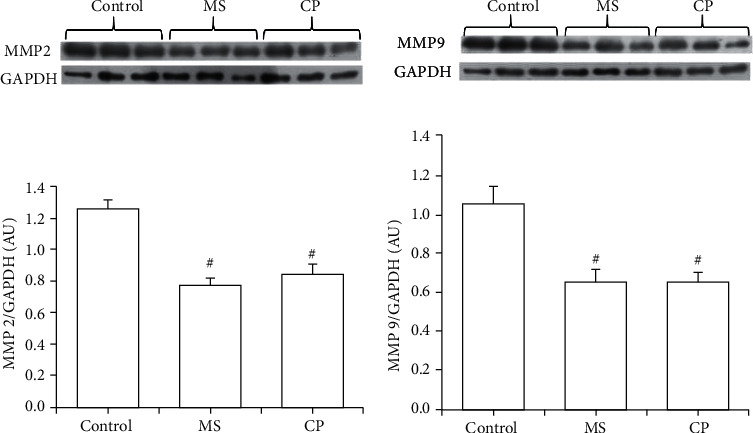
Expression of metalloproteinases 2 and 9 in aortas from control, MS, and CP rats. There is a decrease in their expression in both groups. MS rats ingested sucrose solution during six months; CP animals ingested sucrose solution during postnatal days 12 to 28. Control rats received tap water. A representative blot is shown above the graphics. Values are means ± SEM; ^#^*P* ≤ 0.05; *n* = 8.

**Figure 5:**
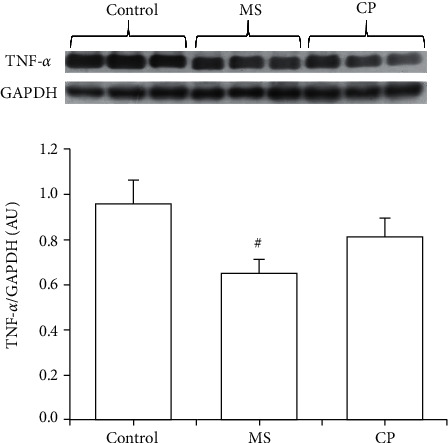
Expression of TNF*α* in aortas from control, MS, and CP rats. MS rats ingested sucrose solution during six months; CP animals received sucrose solution during postnatal days 12 to 28. Control rats received tap water. There is a decrease in their expression in both groups when compared to C animals. A representative blot is shown above the graphic. There is a decrease in TNF*α* expression in MS when compared to C animals. Values are means ± SEM; ^#^*P* ≤ 0.05 versus the control group. *n* = 8.

The expressions of MMP-2 and MMP-9, which are involved in the development of hypertension, were significantly decreased in aortas from CP and MS rats (Figure 4). The expression of TNFα was decreased in the aortas from MS and CP rats (Figure 5).

### 3.4. Vascular Responses

**Figure 6:**
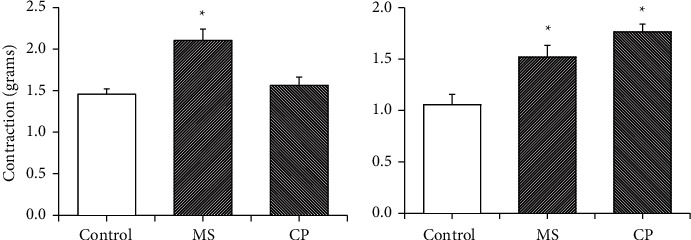
(a) NE-induced contraction (1 *μ*M) and (b) KCl-induced contraction (40 mM) in aortic rings from control rats that received tap water, metabolic syndrome (MS) rats that ingested sucrose solution during six months and critical period, and CPrats that ingested sucrose solution during postnatal days 12 to 28. Concentrations were chosen after performing a dose-response curve in previous studies. Values are means ± SEM; ^*∗*^*P* < 0.01 versus other groups. *n* = 6.

Contractile responses to NE (1 *μ*M) and KCl (40 mmol/L) are shown in Figure 6, panels A and B, respectively. Contraction elicited by NE was stronger than KCl contraction. Vascular response to NE was only increased in MS rats receiving sucrose during six months, and this increase was not present in the CP group. In contrast, there is an increased contractile response to KCl in MS rats and in the CP rats.

**Figure 7:**
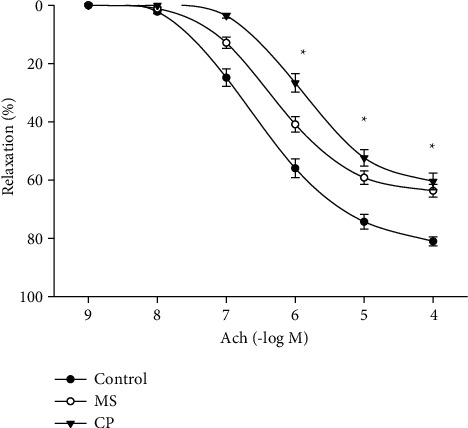
Aortic in vitro relaxation produced by acetylcholine in NE-precontracted aortic rings (1 *μ*M) from control, metabolic syndrome (MS), and critical period (CP) rats. MS rats ingested sucrose solution during six months; CP animals ingested sucrose solution during postnatal days 12 to 28. Control rats received tap water. Values are means ± SEM; ^*∗*^*P* < 0.01 versus the control group.

Vascular relaxation to Ach in NE-precontracted aortic rings was tested and is shown in Figure 7. Control aortic rings showed maximum dilation response *E*_max_ = 81.0 ± 1.6%. Aortic rings from MS rats that received sucrose during and after the CP showed *E*_max_ = 63.7 ± 2.2%. Aortic rings from CP rats that received sucrose only during also showed a decreased relaxing response (*E*_max_ = 60.4 ± 2.8%). The EC50 values from the concentration-response curves of ACh were also evaluated, but no significant differences among the groups were found (3.2 × 10^−7^ ± 6.4 × 10^−8^ mol/L for the control group, 4.1 × 10^−7^ ± 7.3 × 10^−8^ mol/L for the MS group, and 4.9 × 10^−7^ ± 8.2 × 10^−8^ mol/L for the CP group).

**Figure 8:**
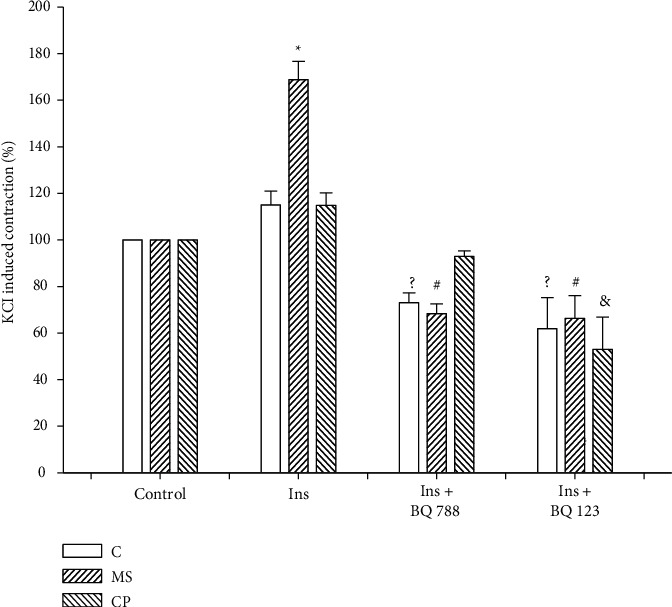
Participation of endothelin and endothelin receptors ET_A_ and ET_B_ (by the use of 1 *μ*M of antagonist BQ123 and BQ788, resp.) in the presence of 50 *μ*U/mL of insulin in KCl (40 mM)-contracted aortic rings. MS: metabolic syndrome rats that ingested sucrose solution during six months; CP: critical period animals that ingested sucrose solution during postnatal days 12 to 28. Control rats received tap water. Values of KCl-induced contraction without insulin were normalized to 100% with respect to values in Figure 6(b) (first set of bars). Values are means ± SEM; ^*∗*^*P* < 0.001 MS versus C Ins and CP Ins; ^?^*P* > 0.001 versus C Ins; ^#^*P* < 0.001 versus MS Ins; ^&^*P* > 0.001 versus CP Ins. *n* = 6.

The participation of endothelin and endothelin receptors was tested in KCl contracted aortic rings in the presence of insulin ET_A_ and ET_B_ by the use of receptor blockers BQ 788 for ET_B_ receptors and BQ123 for ET_A_ receptors. Responses are shown in Figure 8. Values of KCl-induced contraction without insulin were normalized to 100% (first set of bars) with respect to values in Figure 6. Insulin increases the response to KCl in aortas from MS rats but not in control and CP rats. The blockage of endothelin receptors ET_A_ and ET_B_ reduced the contraction of aortas in control rats, even if there was no increase in contraction due to insulin, manifesting the participation of endothelin in contraction. Both receptors completely blocked the increase in contraction induced by insulin in aortas from the MS rats. However, only the BQ123 receptor blocker of ET_A_ receptors decreased the response in CP rats in which there was no insulin-induced increase in contraction, and therefore only the ET_A_ receptors participated in the KCl-induced contraction in this group.

## 4. Discussion

Even well-treated and controlled adults with hypertension have substantial excess mortality and reduced survival compared with normotensive subjects, and therefore, the identification of the means of preventing hypertension since early life is important. Exposure to risk factors in childhood may have long-term influences on vascular structure and function, and essential hypertension may be programmed since childhood [23–29]. The study of critical periods is important since changes occurring at these stages might predispose to the development of diseases in adult life [1] such as glucose intolerance and type 2 diabetes [30] and hypertension, including the high blood pressure due to MS and that induced by sucrose ingestion during the critical window previously described [9].

In this study, metabolic syndrome was established in rats receiving sucrose during 6 months, in accordance with previous studies (Table 1) [12]. We did not find that increased ingestion of sucrose during the critical period increased the incidence of MS during adulthood, and only the presence of hypertension was found [9]. Rats receiving sucrose during this lapse of time showed similar body weight, abdominal fat, glucose, insulin, and triglycerides to control rats. The lack of effect on the development of MS in CP rats might be due to the short-term administration of sucrose used in this study which is shorter than the period of administration used in other papers.

Hypertension may result from structural and functional changes known as vascular remodeling in large conductance vessels such as the aorta and in resistance arteries which include small arterioles (200–30 *μ*m) [1, 31]. Both conductance and resistance vessels contribute to hypertension [1]. In our CP rats, the lumen of the aortas, which are conductance arteries, was decreased, and there was only a tendency to decrease in the MS group (Figure 1 and Table 2). The total area of the media and adventitia was reduced in both groups, possibly leading to changes in the contractility of the vessels. Different types of remodeling may be found in arteries, inward remodeling that denotes a reduction in vessel size, and outward or excentric remodeling that denotes an increase in vessel size. Remodeling may also be hypertrophic and hypotrophic [32]. Our samples showed inward and hypotrophic remodeling. Remodeling in large conductance arteries such as the aorta includes an increase in stiffness, which reduces their capacity to transform the pulsatile pressure to continuous pressure and flow in arterioles with minimal energy. This capacity defines arterial compliance that depends on the intrinsic material stiffness and the arterial geometry [31]. In contrast, to changes in conductance arteries, remodeling in resistance arteries includes media thickening, reduced lumen diameter, and an increased media : lumen ratio and is accompanied by altered VSMC growth, migration, differentiation, and increased extracellular matrix abundance [33]. Changes in resistance arteries correspond to the secretory phenotype of VSMC and are also present during aging [33].

Hypertension is characterized by reduced distensibility and endothelial dysfunction [34]. Vascular stiffness results from fibrosis which was not observed in the aortas from our experimental groups and extracellular matrix remodeling. These processes are amplified by hypertension and are also associated with aging [34]. Although it could be expected that, in hypertension, the total area of the media and adventitia of conductance arteries would be increased, this was not observed in our groups of rats, and instead, the width of the layers was reduced. It is possible that changes in resistance arteries from these rats might compensate for the opposing changes that we found in the aortas and lead to the hypertensive state. It would be important to conduct studies on resistance arteries from these groups.

The changes in the organization of the aortas of the CP and MS aortic histology (Figure 2) might underlie changes in the phenotype of VSMC phenotype and in contractility. One of the markers of the presence of the secretory phenotype in VSMC is a decrease in *α*-actin. In this paper, we found that the expression of *α*-actin was diminished in the aortas from the CP and MS rats (Figure 3). This change suggests a switch of the VSMC to the secretory phenotype in which vasoreactivity is decreased. We also examined the expression of *β*-actin, and it was only decreased in the MS group. The vascular phenotype in a young hypertensive individual is similar to that of an elderly healthy individual, and therefore, the concept of “early” or “premature” vascular aging is employed for hypertension-associated vascular disease [34]. We had previously reported premature aging in vessels from metabolic syndrome rats according to their vasomotricity [35].

The change in the phenotype of VSMC is accompanied by alterations in the activity of MMPs, which are endopeptidases that degrade various proteins in the extracellular matrix, including collagen and elastin. Alterations in specific MMPs, such as MMP-2 and MMP-9, which are gelatinases expressed on cell surfaces (MMP-2) or secreted (MMP-9) by endothelial cells and myofibroblasts influence arterial remodeling, leading to various pathological disorders that include hypertension [36, 37]. In this paper, we determined the expression of MMPs and surprisingly found that MMP-2 and MMP-9 were diminished in CP and MS rats (Figure 4).

Although there is a high expression, secretion, and activation of MMPs in tissues undergoing vascular remodeling, the expression of active MMPs is absent or very low in mature and quiescent vessels [19]. We suspect that the VSMC phenotype was fixed during the critical window and that cells remain in this state of differentiation. Therefore, a steady stationary state outside normal regulation levels of factors involved in VSMC differentiation and in its secretory or contractile phenotype such as MMPs is established and fixed during the CP, and it lasts for a long lapse of time until adulthood. Developmental stability and instability of phenotypic plasticity have been previously described [38]. It would be important to determine the expression of these MMPs at the end of the critical window with and without the administration of sucrose, where the change is active, and their expression might be increased. Decreased vascular MMP-2 and MMP-9 may lead to decreased vasodilation, increased vasoconstriction, hypertensive pregnancy, and preeclampsia [39]. The consequent extracellular matrix rearrangement and phenotype switch of VSMCs lead to increased cellular migration and proliferation [40].

Moreover, transcriptional regulation of MMPs is tissue-specific and depends on a large number of signaling pathways and specific transcription factors (such as NF-*κ*B, AP-1, CREB, MAP kinases, and the Smad family of proteins). It may also be determined by epigenetic control [41]. When these signaling pathways are blocked, there is a decrease in the synthesis of some downstream mediators, transcription factors may be kidnapped, preventing their binding, or their phosphorylation may be inhibited, thus repressing the expression of MMPs [42]. It would therefore be important to evaluate which of these factors could be involved in the downregulation of the expression of the MMPs analyzed in this paper. Another possible explanation for the decreased expression of MMP-2 and 9 may be that although their expression is decreased, their activity might be increased. Exploration of some downstream targets of MMPs such as collagen 4 or elastin might shed light on the activity of these enzymes in these models, and the absence of data in this respect constitutes a limitation of this paper. Furthermore, the balance between the expression and activity of MMPs and that of the tissue inhibitors of these enzymes (TIMPs) should also be studied, and the lack of data on the activity of TIMPs is a limitation of this study.

Another factor contributing to vascular remodeling is inflammation, which is linked to macrophage infiltration, fibrosis, and increased expression of redox-sensitive proinflammatory genes [31]. TNF*α* also has profound effects on VSMCs, including the change from a contractile to a secretory phenotype. This switching promotes the proliferation and production of extracellular matrix proteins, which are associated with medial hypertrophy [43]. In this paper, we found that the expression of TNF*α* was decreased in CP and MS rats (Figure 5) which coincides with the diminution in the expression of MMPs, which also contribute to inflammation (Figure 5). There is a previous report indicating that the serum level of TNF*α* was not decreased in MS rats [44] even when inflammation was present. It is possible that local TNF*α* and MMPs were decreased in this study since inflammation was present during the first stages of the sucrose ingestion in MS rats and during its ingestion during the CP; however, compensatory mechanisms might have initiated and persisted during the following months, which normalized and even decreased their levels.

Hypertension is usually linked to changes in arterial contractility. In this paper, we studied vascular contractility of aortas from rats with hypertension derived from MS and CP in vitro. These changes reflect endothelial dysfunction. There had been previous reports of enhanced contractility in rats with MS and in aortas in the presence of high insulin and glucose concentrations [45], and therefore, we previously studied the contractility of aortas during the CP of the pancreas where there are important changes in plasma concentrations of glucose and insulin [11]. Here, we analyzed the KCl- and NE-induced contractions. KCl depolarizes the cellular membrane of smooth muscle cells allowing calcium into the cytoplasm and activating the contractile machinery, and therefore, contraction in the presence of KCl shows the response of the artery when both the endothelial cells and VSMC are depolarized. The sensitivity of the myofilaments to Ca^2+^ changes during development in the agonist-induced contraction of vascular smooth muscle [46]. When depolarized by potassium, vessels are less sensitive to calcium in younger animals [47]. The KCl dose used in our previous and our present paper is a submaximal dose, which has been previously chosen to test insulin-induced changes in contractility in a previous paper by our group [48].

NE-induced contraction involves the liberation of vasoactive substances by the endothelium. Alpha-adrenergic receptors are G protein-coupled receptors and mediate some of the physiological actions of NE. These receptors activate a variety of effectors, including phospholipase C (PLC), phospholipase D, phospholipase A2, cAMP metabolism, and several ion channels. The activation of phospholipase A2 leads to the liberation of arachidonic acid, which may be metabolized by cyclooxygenases to produce metabolites that modify vascular responses [49]. The contraction that results from administration of NE shows the response of VSMC induced by the endothelium, in contrast to the contraction elicited by KCl. Maturation and aging are associated with many alterations in vascular adrenergic mechanisms [50]. From birth to adulthood (maturation) and from adulthood to old age (aging or senescence), important changes occur in animal models as in humans at the receptor level, neurotransmitter process, and catecholamine inactivation. In general terms, maturation is associated with an increase, whereas aging is associated with a reduction in the adrenergic influence on the physiological processes [50]. In our study of the variations in aortic contraction during the CP, we found that contraction elicited by NE was stronger than KCl contraction in all groups (Figure 6). In a previous paper, we described that NE-induced contraction increased from day 12 to day 28 but stabilized from day 21 to day 28. It was stronger in controls and increased in MS rats [11]. In the present paper on the effects of a change in diet during this lapse of time on the development of hypertension in the adult, we found that it did not increase the contractile response since an elevation was only found in the aortas from the MS group.

We had previously described that KCl-induced contraction increased with age during the CP being weaker than in C rats and that it further increased in MS rats. In the present study, we found that KCl-induced contraction was stronger in MS rats than in controls and that the treatment with sucrose both during the CP significantly increased the force of contraction [11]. Therefore, changes in sucrose diet might alter the liberation of calcium from the sarcoplasmic reticulum or the sensitivity of myofilaments to calcium but not the liberation of vasoactive substances from the endothelium, but more research is needed to clarify this issue. There could also be a change in the phenotype of VSMC from the contractile form to the secretory form [13].

It has been previously reported that endothelium-dependent vasorelaxation is significantly reduced in the MS model in which rats receive sucrose during and after the critical window of the pancreas [11]. Groups of rats receiving sucrose only during the CP also showed a reduced Ach-induced relaxation (Figure 7). Acetylcholine induces relaxation by increasing the synthesis and release of NO, and nitric oxide synthases are a target of epigenetic control [51]. The activity of eNOS is depressed when sucrose is ingested during the critical window [9]. We had previously reported that vasorelaxation to Ach in NE-precontracted rings did not change during the neonatal period being similar to that in MS rats and lower than that in controls. If the synthetic phenotype opposes the recovery of the vasorelaxation of the aortic ring from CP, it would have been important to assay the effect of an NO donor on vascular muscle directly without the endothelium, which is lacking in this study and is a limitation of this study.

Insulin increases KCl-induced aortic contraction through changes in endothelin release, and therefore, the role of endothelin can be studied in aortic rings contracted in the presence of insulin and endothelin receptor antagonists [22]. Serum endothelin levels were increased in the MS and CP groups. Endothelin is an autocrine and paracrine factor whose activity is difficult to measure in vivo [52]. Insulin-induced increase in contraction is mediated by endothelin receptors ET_A_ and ET_B_ [45]. Although ET_A_ receptors, which are predominantly located on smooth muscle cells, function to promote vasoconstriction, growth, and inflammation, while ET_B_ receptors, mainly located in endothelial cells, produce vasodilation and inhibit growth and inflammation, results are contradictory and complicated to interpret [52]. In this paper, we tested the insulin increase in KCl-induced contractility in our different experimental groups. In the MS, there was a previously reported increase in contractility. This increase was not observed in the rat aortas from the control and CP rats. Both receptor blockers (BQ788 that blocks ET_B_ and BQ123 that blocks ET_A_) inhibited the insulin response in MS aortas, and only the ET_B_ receptors were involved in the tendency to an increase in contractility induced by insulin in the CP rats since they blocked the response elicited by insulin. Two different pathways are activated in vasoreactivity changes produced by insulin: the phosphatidyl inositol 3 kinase (PI3K)/protein kinase B (PKB or AKT)/eNOS and the mitogen-activated protein kinase (MAPK)/extracellular signal-regulated kinase (ERK) pathways. PKB phosphorylates eNOS increasing NO production and vasodilation, while the MAPK pathway results in endothelin-1 production and vasoconstriction [53] and may be participating in the responses found in this paper.

## 5. Summary of Results and Conclusion

In summary, a high sucrose intake during the critical window increases arterial blood pressure when individuals reach adulthood. Alterations in the variables associated with MS are smaller in CP rats due to the limitation in time of exposure to sucrose. There was an increased lumen of the aortas in CP and MS rats and reduced media and adventitia. The VSMC phenotype was changed to a secretory phenotype, but MMP-2 and MMP-9 were reduced. The effect on blood pressure is mainly due to reduced vasorelaxation. In conclusion, it is important to control diet during the early stages of development to reduce the risk of developing hypertension in adults. How a high sucrose diet during the critical window of the pancreas could affect early programming leading to hypertension is unclear.

## Figures and Tables

**Table 1:** Body and biochemical variables in the control, MS, and CP groups.

	Control	MS	CP
Body weight (g)	536.4 ± 12.0	546.1 ± 24.4	540.7 ± 12.5
Central adiposity (g)	5.4 ± 0.4	11.4 ± 0.5^*∗*^	6.1 ± 0.8
Blood pressure (mm of Hg)	101.9 ± 1.4	135.4 ± 2.9^∗∗^	138.9 ± 0.8^∗∗^
Glucose (mM)	5.6 ± 0.2	5.5 ± 0.1	5.4 ± 0.2
Insulin (*μ*U/mL)	3.13 ± 0.8	7.1 ± 0.8^*∗*#^	1.9 ± 0.4
HOMA index	0.9 ± 0.1	2.3 ± 0.2^&^	1.9 ± 0.2
Triglycerides (mg/dL)	64.3 ± 2.7	125.7 ± 11.9^†^	61.4 ± 3.6
ET-1 (pmoles/mL)	2.1 ± 0.3	4.7 ± 0.7^#^	3.3 ± 0.4^#^

Values are mean ± SEM *n* = 8, ^*∗*^*P* < 0.01 MS versus C and CP; ^*∗∗*^*P* < 0.001 MS and CP versus C; ^#^*P* < 0.05 MS and CP versus C; ^†^*P* < 0.01 MS versus C and CP; ^&^*P* < 0.05 MS versus C and CP.

**Table 2:** Area of the lumen, the media (Med), and the adventitia (Adv) and width of the layers of aortas from control, MS, and CP rats.

	Control	MS	CP
Total area (pixels^2^)			
Lumen	13.10 + 0.39	11.53 + 0.55	5.62 + 0.28^*∗*^
Media	4.02 + 0.11	2.81 + 0.14^*∗*^	2.33 + 0.08^*∗*^
Adventitia	2.91 + 0.12	1.98 + 0.10^*∗*^	1.02 + 0.06^*∗*^
Width (pixels)			
Media	82.46 + 6.23	20.86 + 2.31^*∗*^	26.39 + 2.54^*∗*^
Adventitia	73.49 + 14.91	12.36 + 2.76^*∗*^	10.91 + 3.03^*∗*^

MS: metabolic syndrome rats that ingested sucrose solution during six months; CP: critical period animals that ingested sucrose solution during postnatal days 12 to 28. Control rats received tap water. Values are mean + SEM; *n* = 12 images (at least 3 images for each animal (4) for each group). Values are mean + SEM; *n* = 12. The area is expressed as pixels^2^ × 10^5^. ^*∗*^*P* ≤ 0.01 with respect to controls.

## Data Availability

Data are available from the corresponding author upon request.
